# What are the factors affecting parents’ postnatal sense of security?

**DOI:** 10.18332/ejm/140139

**Published:** 2021-09-08

**Authors:** Vesile Koçak, Eva-Kristina Persson, Elizabeth Crang Svalenius, Kamile Altuntuğ, Emel Ege

**Affiliations:** 1 Department of Obstetric and Gynecology Nursing, Faculty of Nursing, Necmettin Erbakan University, Konya, Turkey; 2 Department of Health Sciences, Faculty of Medicine, Lund University, Lund, Sweden

**Keywords:** parents, postpartum period, care, postnanal sense of security

## Abstract

**INTRODUCTION:**

The postpartum period is part of an important process for mothers and fathers. A sense of security is central as it might influence a parent’s journey towards becoming a successful parent. The aim was to determine factors affecting parents’ postnatal sense of security (PPSS) before postpartum discharge from a hospital in Konya, Turkey.

**METHODS:**

A descriptive study was conducted. From January 2019 to March 2019, a questionnaire was given to a convenience sample of 188 couples discharged from a regional hospital in Turkey. The sense of security was assessed using the PPSS instrument, with low scores defined as those less than the mean.

**RESULTS:**

Low and high sense of security was based on the mean in the population, for mothers 49.36 and for fathers 34.90. It was found that 43.6% of mothers and 69.7 % of fathers had a low score, which was linked to some specific factors in the postpartum period. These were the type of birth, being ready to take responsibility for baby care, being ready to be discharged, being healthy, having any concern about the baby’s health, social support presence, having professional support, and presence of a sense of security.

**CONCLUSIONS:**

Many parents, particularly fathers, have a low postnatal sense of security. In the postpartum period, it is very important for midwives, who are always with the family, to identify the risks for a low sense of security during this period and provide effective care. More studies in different settings with larger samples are recommended.

## INTRODUCTION

Becoming a parent is a significant life transition that can be confusing and overwhelming when feelings of joy are mixed with the strain of parental responsibilities^1,2^. The transition to parenthood can bring many changes during this important life event^3^. Following childbirth, parents report physical and emotional changes^4^. Being prepared, having knowledge and receiving social and professional support may facilitate this transition^1^. Parents’ experiences of the early parenthood period are affected by the information that they are given before birth about the early postnatal period^5,6^. The early parenthood period can be a time of insecurity when parents strive for confidence^7^. A sense of security is important during the first postnatal weeks, for parents as individuals, as a couple, for their start as parents and the baby’s wellbeing^8^. It is therefore important to study and understand both parents’ sense of security and their needs during the transition to parenthood in the immediate postpartum period.

A sense of security is a central element to support as it might influence a parent’s journey towards becoming a successful parent. Persson et al.^8^ developed the concept of ‘parents' postnatal sense of security (PPSS)’. They identified in their Swedish study the following dimensions as important for both parents’ postnatal sense of security: empowerment from staff, affinity within the family, and the health and wellbeing of the family. An empowering organization was fundamental for strengthening this. In Sweden, fathers are encouraged to participate in all aspects of pregnancy, delivery and the postnatal period^8^. If parents feel insecure in the postpartum period, this feeling may turn into anxiety and depression in the future^8,9^. A study found that depressive symptoms in the early postpartum period are 5–20% in mothers and 3–10% in fathers^10^. According to The World Health Organization, depression can be reliably diagnosed and treated in primary care settings^11^. Transition to parenthood represents a significant life event increasing the vulnerability to psychological disorders. Health professionals have an important responsibility to provide the parents with postpartum care by enhancing and supporting their sense of security^12^.

The postpartum period is of great importance for mother and newborn due to the physiological and psychological changes experienced by the mother and changes in family structure^13^. The assessments after birth should include a broader assessment of life’s difficulties^14^. The nurse/midwife should collaborate with the mother toward independence and fulfilment, which can contribute to improving neonatal and maternal outcomes^14^.

Fathers are at increased risk of mental health problems in the transition to fatherhood, too; transition to parenthood involves the father as much as the mother, and requires adjustment and reconstruction of behavior for the new role^15^. Although mothers undertake most parental activities, there is an increasing acknowledgement that fathers also play a vital role, but the available literature primarily focuses on the study of maternal role with little on that of the fathers’^16^. According to Feenstra et al.^15^, fathers are the practical guy in the shadow in the early postnatal period, the fathers in the shadow of the mothers. When they are at home for the first days, they experience ups and downs in the early postnatal period, where they do not feel prepared for the challenges. Their focus is on the mother’s and newborn’s needs and not their own. But at the same time, they view themselves as equal to the mother in respect to the parental role^16^. Despite a focus on the family-centered approach, studies still demonstrate that healthcare professionals are unsuccessful at involving fathers in antenatal and postnatal care^17^. Expectant and new fathers wish to receive information about the mother’s and newborn’s health and be properly prepared for early discharge^18,19^. Nevertheless, the fathers report frustration about the absence of father-focused sessions pertinent to postpartum issues and skills. Many fathers report struggling due to a lack of knowledge, expressing disappointment with the lack of information or programs targeted specifically for fathers^20^.

According to the TNSA 2018 (Turkey Demographic and Health Survey 2018)^21^ results, 96% of mothers in Turkey received care from health personnel before birth. After birth, 79% of women received postpartum care in medical check-ups, including assessment of the perineum and breastfeeding. In the postpartum period, 66% of Turkish mothers receive postpartum care from healthcare personnel in the first four hours after birth, and 83.4% in the first two days after birth^21^. Professionals should view early parenthood as a joint project and support both parents’ needs^19^. Nurses and midwives are, in Turkey, the primary caregivers together with doctors during the postnatal care period. It is very important that the nurses/midwives, who are always with the family in the postpartum period, ascertain the problems and provide effective care.

The aim of this study was to determine factors affecting parents’ postnatal sense of security before postpartum discharge from a hospital in Konya, Turkey.

## METHODS

### Study participants

A total of 188 married couples who were at the postpartum unit and who were willing to participate in the study were recruited. Inclusion criteria were: aged >18 years, having a healthy baby, and both parents willing to participate in the study. The exclusion criterion was an incomplete response to the questionnaire. The participants were visited by a researcher (VK) at the postnatal department during visitors’ hours to find them together. A convenience sampling method was used in the study. The method was chosen because of easy access to parents.

### Design and settings

The population of the study comprised both first time and multiparous parents in the postpartum unit of a hospital in Konya, Turkey, with approximately 1800 births a year. There are 12 rooms at the department. Two are private rooms and the others are for two people. Mothers who give birth can stay with the newborn at the department. If the room is a private room, the father can stay with the mother and baby for the whole day. Otherwise, a female companion, who stays with the mother, helps her; male presence is considered out of place. This female companion generally is a mother, mother-in-law, or sister. For this reason, the father participants were at the department only during visiting hours (13:00–15:00). In Turkey, mothers and newborns are discharged 24 hours after normal deliveries and 48 hours after cesarean deliveries, if they are healthy. Turkey’s Health Ministry and especially the ‘mother-friendly hospitals’ want to increase the number of normal births and also want to introduce an approach that does not allow cesarean section except for medical reasons. Fathers are now attending childbirth classes with mothers. However, it is not possible for fathers to easily attend the birth in all public hospitals. Depending on the hospital policies, fathers also are ignored during the postpartum period. However, there are positive legal developments in Turkey that support the participation of fathers in the postpartum process. For the father’s support to the mother postpartum and in order to participate in baby care, Law No. 657 on Civil Servants has been amended. According to this law, fathers can get 5–10 days paid paternity leave.

This research was conducted at a postpartum unit at the university hospital in Konya where approximately 1800 women give birth each year. The data collection process was conducted from January 2019 to March 2019. The postpartum units provide medical services, support, and education to mothers with their newborns. The hospital where the study was carried out was preferred because it was the largest university hospital in the city. The data were collected when the mother and baby had been hospitalized in the postpartum department for 12 to 48 hours after delivery. Data from the fathers were collected when they were at the hospital, visiting the mother and baby after delivery. Some fathers were with mothers who were in a private room; some of the fathers at a place for visitors. A researcher (VK) visited all of them when they were available to answer the questionnaire. The data were collected in the presence of the researcher.

### Questionnaire

A questionnaire was used to explore parents’ postnatal sense of security before hospital discharge, measured with the PPSS instrument. Information was collected using a questionnaire for the descriptive and obstetric characteristics of the participants and the PPSS instrument^8^. Before filling in the questionnaire, the parents were asked to read and approve the informed consent form explaining the purpose of the study, if they were willing to participate.

### Sociodemographic and background questions

Sociodemographic characteristics and background questionnaire, which was developed by the researchers based on literature, consists of 17 questions focused on the participants’ sociodemographic characteristics and topics related to obstetrics and the postpartum period. The sociodemographic questions addressed age, education level, employment, economic status, and family type. Background questions about pregnancy, delivery and the postpartum period addressed the planning for the pregnancy by the woman and her spouse, type of birth, baby’s gender, previous living children, having education about the postpartum period, having responsibility in baby care, being ready to be discharged, having concern about the health of mother and baby, having social support, having professional support, and feeling a sense of security. The questionnaire was pilot tested for understanding with ten couples who were not included in the sample. After the pilot, the order of the questions was revised.

### Instrument

Parents’ postnatal sense of security (PPSS) instrument^8^ was used for data collection. The mothers’ version of the PPSS instrument was tested for validity and reliability in Turkey by Geçkil et al.^22^. The instrument measures the mother’s sense of security during the first week postpartum. It is a 4-point Likert scale with 18 items (1=Disagree, 2=Agree somewhat, 3=Agree quite a lot, 4=Strongly agree). Scores taken from the instrument ranged between 18–72, and high scores indicate a high sense of security. The Turkish version of the PPSS instrument for mothers has a Cronbach’s alpha coefficient of 0.84 and is valid and reliable^22^. In the Persson et al.^8^ study, the mean score for women on the PPSS instrument was 56.3 (range: 18–72), and the Cronbach’s alpha coefficient was 0.88. The higher the score, the greater the individual’s feelings of security^8^.

The father’s version of the PPSS instrument was tested for Turkish validity and reliability by Koçak et al.^23^. The scale measures the father’s sense of security in the first week after birth. It consists of 13 items, on a 4-point Likert scale (1=Do not agree at all, 2=Agree a little, 3=Agree quite a lot, 4=Totally agree). The scores obtained from the instrument varied between 13 and 52, and high scores indicate that their sense of security is good. Cronbach’s alpha coefficient of the instrument was 0.74^23^. In the study of Persson et al.^8^, from the south of Sweden, the mean score for fathers on the PPSS instrument was 40.4 (range: 13–52) and Cronbach’s alpha coefficient was 0.77.

### Data analysis

Data analysis was carried out using Statistical Package For Social Science (SPSS) 20.0 package program. A p<0.05 was considered statistically significant. Descriptive statistics, including frequency, means and standard deviations, were calculated. The Kolmogorov-Smirnov test was performed to test whether the data were normally distributed. An independent sample t-test was also performed as the PPSS scores showed normal distribution. The low and high scores were calculated according to the cut-off point mean scores of participants’ PPSS scores. Independent samples t-test was used to examine differences in obstetric and postpartum characteristics of the parents between the postpartum sense of security scale mean score.

### Ethical considerations

All procedures performed in studies involving human participants were in accordance with the ethical standards of the institutional and/or national research committee and with the 1964 Helsinki declaration [World Medical Association (WMA) 2018]^24^ and its later amendments or comparable ethical standards. The participants were informed about the study both verbally and in writing and were included after giving their informed consent in compliance with the Helsinki Declaration^24^. This study was approved by The Ethics Committee for Scientific Research and Publication in Medicine Faculty at Necmettin Erbakan University (2017/1016-22.09.2017).

## RESULTS

### Sociodemographic characteristics

Table 1 shows the sociodemographic distribution of the participants. Participants’ age, education level, employment, economic status and family type are presented.

**Table 1:** Sociodemographic characteristics of parents (N=188)

*Characteristics*	*Mother Mean±SD*	*Father Mean±SD*
**Age** (years)	29.48±5.46	33.27±5.71
	***n (%)***	***n (%)***
**Education level**		
Primary	90 (47.9)	64 (34)
High school	63 (33.5)	62 (33)
University	35 (18.6)	62 (33)
**Employment**		
Employed	134 (28.7)	187 (99.5)
No employment outside the home	54 (71.3)	1 (0.5) (unemployed)
**Perceived economic status**		
Good	62 (33.0)	59 (31.4)
Moderate	111 (59.0)	126 (67.0)
Bad	15 (8.0)	3 (1.6)
**Family type**		
Nuclear	153 (81.4)	153 (81.4)
Extended	35 (18.6)	35 (18.6)

### Parents’ postnatal sense of security

The mean score of the postpartum sense of security for mothers was calculated as 49.36±12.84, the mean score of the fathers as 34.90±9.57. According to the mean scores in this study, among the mothers, 43.6% obtained a low score of PPSS and 56.4% obtained a high score. Among the fathers, 69.7% obtained a low score of PPSS and 30.3% a high score (Table 2). None of the sociodemographic variables showed significant differences in relation to the postnatal sense of security, either for mothers or fathers.

**Table 2:** Parents’ postpartum sense of security (PPSS) mean score

*PPSS score*	*Mother n (%)*	*Father n (%)*	*Mother Mean±SD*	*Father Mean±SD*
Low	82 (43.6)	131 (69.7)	49.36±12.84	34.90±9.57
High	106 (56.4)	57 (30.3)		

### Parents’ postnatal sense of security in relation to background questions about pregnancy, delivery and the postpartum period

There is a significant difference in the parents’ postnatal sense of security in relation to the type of birth. Parents who had their baby by vaginal delivery had a higher sense of security (p<0.05). Being ready to take responsibility in baby care makes a difference for the sense of security of parents. Parents who felt ready to take responsibility for baby care also had a high postnatal sense of security score (p<0.05). It was found that there is a significant difference in parents’ postnatal sense of security between being ready to be discharged. Parents who were feeling ready to be discharged felt more secure (p<0.05). Parents who had any concern about the mother’s health and the baby’s health felt more insecure (p<0.05). The availability of social and professional support made a significant difference in the postpartum sense of security score. Parents who had social support and professional support felt more secure (p<0.05). The baby’s gender made a difference in mothers’ postpartum sense of security. Mothers who had female babies had a lower sense of security than fathers (p<0.05). For fathers, the baby’s gender was irrelevant (p>0.05) (Table 3).

**Table 3:** Obstetric and postnatal characteristics in relation to parents’ postnatal sense of security scale mean score

*Characteristics*	*Mother*	*Father*	*Mother*		*Father*	
	*n (%)*	*n (%)*	*Mean±SD*	*p*	*Mean±SD*	*p*
**Planned pregnancy**						
Planned	165 (87.8)	167 (88.8)	49.32±13.01	0.922	35.23±9.66	0.176
Unplanned	23 (12.2)	21 (11.2)	49.60±11.86		32.23±8.56	
**Type of birth**						
Vaginal delivery (including instrumental deliveries)	62 (33.0)	62 (33.0)	55.29±10.44	**0.000**	40.14±7.66	**0.000**
Cesarian section	126 (67.0)	126 (67.0)	46.44±12.95		32.32±9.39	
**Baby’s gender**						
Female	87 (46.3)	87 (46.3)	46.87±14.22	**0.015**	33.95±10.39	0.208
Male	101 (53.7)	101 (53.7)	51.50±11.16		35.72±8.78	
**Previous living children**						
0	77 (41.0)	77 (41.0)	49.38±13.27	0.980	33.98±9.75	0.275
≥1	111 (59.0)	111 (59.0)	49.34±12.60		35.54±9.43	
**Education about the postpartum period**						
Yes	49 (26.1)	20 (10.6)	51.91±10.98	0.105	37.55±9.59	0.192
No	139 (73.9)	168 (89.4)	48.46±13.36		34.58±9.55	
**Responsibility for baby care**						
Ready	162 (86.2)	164 (87.2)	50.53±12.25	**0.002**	35.89±8.91	**0.003**
Feeling ready to be	26 (13.8)	24 (12.8)	42.03±14.27		28.16±11.32	
**Feeling ready to be discharged**						
Yes	154 (81.9)	162 (86.2)	51.33±12.13	**0.000**	35.87±9.16	**0.000**
No	34 (18.1)	26 (13.8)	40.41±12.31		28.84±10.00	
**Concerned for own health** (mothers’ health for fathers)						
Yes	39 (20.7)	61 (32.4)	38.43±15.47	**0.000**	27.27±9.46	**0.000**
No	149 (79.3)	127 (67.6)	52.22±10.36		38.56±7.19	
**Concerned about baby’s health**						
Yes	49 (26.1)	73 (38.8)	40.18±14.64	**0.000**	27.94±9.15	**0.000**
No	139 (73.9)	115 (61.2)	52.59±10.42		39.32±6.84	
**Social support available**						
Yes	121 (64.4)	117 (62.2)	54.07±9.44	**0.000**	38.13±7.52	**0.000**
No	67 (35.6)	71 (37.8)	40.85±13.83		29.57±10.23	
**Professional support available**						
Yes	71 (37.8)	72 (38.3)	56.22±10.23	**0.000**	39.18±7.78	**0.000**
No	117 (62.2)	116 (61.7)	45.19±12.51		32.25±9.64	
**Feeling a sense of security**						
Yes	149 (79.3)	141 (75.0)	52.88±10.06	**0.000**	37.53±8.04	**0.000**
No	39 (20.7)	47 (25.0)	35.89±13.52		27.00±9.51	

Independent samples t-test, p<0.05 indicates statistical significance.

## DISCUSSION

This study explored the parent postpartum sense of security and identified which factors may affect both parents’ postpartum sense of security. Based on our research, we have found that many of the Turkish parents have a low sense of security in the postpartum period. The mean score of the postpartum sense of security of mothers was 49.4, the mean score for the fathers was 34.9. In the Persson et al.^8^ study, the mean score for women on the PPSS was 56.3 and the mean score for fathers was 40.4. An explanation for the differences can be due to more cesarean sections in Konya. The result shows that fathers felt more insecure than mothers. According to mean scores, in this study, 43.6% of the mothers obtained a low score on the PPSS instrument and 56.4% reported a high score. Among the fathers, 69.7% obtained a low score on the PPSS instrument and 30.3% a high score. Fathers had a lower score in general. The differences may be relevant to the healthcare system. Fathers are not allowed to participate in the whole process of childbirth in the hospital, in contrast to Sweden^5,6,8^, which is considered a possible reason why fathers’ sense of security is lower in Konya, Turkey. Sometimes fathers are not admitted to the delivery room, another reason why they might feel insecure.

One important variable for the sense of security was shown to be the type of birth. There was a noticeable difference in the sense of security between cesarean section or vaginal birth, for both mothers and fathers. Parents who had their baby by cesarean section had a lower sense of security. A cesarean section is a life-saving surgical procedure when complications arise during labor. However, it may cause maternal and perinatal complications^25^. It is considered that a cesarean section, which can have many complications, may cause insecurity. Similarly, studies from Sweden have found that a father whose wife underwent a cesarean section was more insecure^19,26^. Also, Werner-Bierwisch et al.^12^ showed in their review that parents’ perception of security is related to the type of birth. In Sweden about 17% of women give birth by cesarean section^27^ and in Germany about 29.6%^28^. Cesarean sections have been increasingly overused almost everywhere, both in high- and low-income countries. In Turkey, the proportion of births with cesarean sections in live births was 54.9% in 2018, one of the highest rates in the world. Nurses/ midwives should know that emotional problems in the postpartum period may vary according to the birth type^29^ As the cesarean rate is high in Turkey and that a cesarean section is a clear factor for insecurity, nurses/midwives should be aware of this when providing care after a cesarean section birth.

Mothers having concern about their own and the infant’s health, and although they are healthy, having concern about the baby and mother’s health for fathers, induces insecurity in both. This result is similar to earlier research where mothers wish to be met with encouragement and positive confirmation that everything is well^5,6^. Werner-Bierwisch et al.^12^ showed that fathers were having thoughts about medical complications rather than having complications. Other research has also shown that insecurity was associated with the lack of knowledge and skills experienced by the parent, particularly in relation to the infant^30,31^. Based on these results, it is important to be sure of the health status of baby and mother for parents to feel secure. It is more beneficial to provide the information about the situation needed by parents. Rather than simply providing information, it is more important to assess whether this information is adequate or not. Nurses/midwives should be observant of the feedback from parents and should have effective communication techniques and empathy skills.

It has been found that 18.1% of mothers and 13.8% of fathers were not feeling ready for discharge, and there is a difference in parents’ postnatal sense of security between being prepared to be discharged or not. Not being ready to be discharged in the early postpartum period may cause insecurity in parents. Assessment of families and their discharge readiness during this period are important factors for nurses/midwives^32,33^. In the postpartum period, it is necessary that information before discharge is given for the parents to trust their knowledge and feelings after discharge^34^. Input from staff postnatally is shown to be an important part of parental preparation, which leads to a greater sense of security^6,19^. Both parents need more information about practical skills, emotional support and follow-up counseling after discharge under any circumstance^35^.

The availability of social and professional support makes a significant difference in the PPSS score. Parents who had social help and professional support felt more secure. In Turkey, most women can reach the care system in the form of medical check-ups in the first two days^21^. However, there are no precise data on the continuity and quality of follow-up care^36^. Even though there is some support (educational classes, follow-up programs etc.) for mothers, there is not much for fathers. In our study, fathers were much more insecure than mothers. Studies have shown that many fathers perceived that their needs were unimportant; fathers report being excluded, without having their learning needs met and also frustrated by healthcare professionals’ presumptions that they are inefficient caregivers^20^. Also, fathers report frustration about the absence of father-focused sessions pertinent to postpartum issues and skills. Many fathers report struggling due to a lack of knowledge, expressing disappointment with the lack of information or programs explicitly targeted for them^20^. Considering examples in other countries, it seems necessary to organize Konya, Turkey, for more support for fathers during birth and the postpartum period. Postpartum care and consultation should include a 24/7 support service for both mothers and fathers, primarily focused on mental health and not only obstetric follow-up.

When fathers are not supported, the wellbeing of the entire family is at risk. By supporting fathers as individuals, the fathering role might be strengthened^6^. To improve parental role competence and satisfaction, healthcare professionals should develop strategies that impact the whole family and not just a single individual. Supportive parenting programs should be implemented for both mothers and fathers^16^. A true family perspective should be applied in postnatal care, and the new parents viewed as a family unit, not only as medical cases^18^.

Mothers who had a male baby had a higher sense of security. A male child is seen as the person who will continue the family lineage, and it is thought that having a boy increases the status and value of the mother in Turkish society^37^. In this respect, for mothers to have a male baby is more desirable, and they feel more secure about this. It is difficult to be sure about the reason; therefore, more research is needed. For fathers, gender seems not to be necessary for the sense of security.

It appears that studies should be carried out to ensure that both mothers and, especially, fathers receive more social and professional support in Konya, not only medical follow-up. It is deemed necessary to maintain care and support, not only in the hospital but also at home. Support should be with evidence-based information and specific for the individual. The information exchange process between nurse/midwives and parents should be a two-way communication and continue at home. In order for mothers and fathers to feel secure, it is very important that nurses/ midwives support them throughout the whole process.

### Strengths and limitations

Mothers can stay at the hospital only 24 or 48 hours after birth. This necessitated an early time for the research data collection. The original PPSS instrument was developed with a questionnaire given 8 weeks after birth with questions regarding the first postpartum week. This can have affected the different means in the two studies where the Swedish data were retrospective and the Turkish concurrent. This fact also made it difficult to compare means which can help explain differences in low and high sense of security. A limitation of this study was that it was carried out at a single hospital. The strength of the study was that a validated instrument was used in the study, and the PPSS is validated and reliable for the Turkish language. Another strength is that both parents were interviewed.

## CONCLUSIONS

There are many factors influencing postnatal sense of security of mothers and fathers. These need to be addressed during the pregnancy and the postpartum period, both in hospital and after discharge. As vaginal delivery is related to a higher sense of security, it is important to support higher rates of vaginal births. The results also show that changes such as clear evidence-based information and education about baby care is needed. Parents must be prepared for going home, so they will be ready to be discharged. Both parents must be reassured of the mother’s health and the baby’s health, which a number of parents felt was lacking. Availability of social and professional support before and after birth is important. Change of attitudes to baby’s gender is needed.

It is of importance to have both support and continuity of care. The care should include all family members, both in preparation for birth and for support afterwards. It is important to ask about what parents want to learn about the postpartum period, how they want support, by whom and when they want to be discharged. Before discharge from the hospital, it may be helpful to measure the parents’ postnatal sense of security. Supporting mothers and fathers who do not feel secure enough to be discharged from the hospital, offering them support at home may be helpful. In the postpartum period, nurses/midwives, who are always with the family, can identify risk factors and provide adequate care to parents to strengthen the postnatal sense of security. More postnatal mental and social support is needed. Health professionals can enhance the role of the father by accepting and listening to the father as an individual and take into account the father’s desire to be part of the family from the very beginning. By changing hospital policies, fathers can be supported before, during and after birth. Nurse/midwives are the important providers of healthcare that may contribute to organizing procedures at hospitals. This study was conducted in one hospital in Konya, Turkey. More understanding of the factors that affect the parents’ postnatal sense of security in different settings with larger samples is needed.

## Data Availability

The data supporting this research are available from the authors on reasonable request.
